# Human urinary kallidinogenase combined with edaravone in treating acute ischemic stroke patients: A meta‐analysis

**DOI:** 10.1002/brb3.2431

**Published:** 2021-11-22

**Authors:** Di‐Xiao Yang, Yao Li, Dan Yu, Bi Guan, Qian Ming, Yan Li, Li‐Qing Chen

**Affiliations:** ^1^ Department of Nursing Administration Chengdu Fifth People's Hospital Chengdu China; ^2^ Intensive Care Unit Chengdu Fifth People's Hospital Chengdu China; ^3^ Department of Otorhinolaryngologic Chengdu Fifth People's Hospital Chengdu China

**Keywords:** acute ischemic stroke, edaravone, human urinary kallidinogenase, meta‐analysis

## Abstract

**Introduction:**

Several studies have investigated the efficacy of human urinary kallidinogenase (HUK) combined with edaravone (Eda) in acute ischemic stroke (AIS) patients. Our aim was to provide the best available evidence for clinical practice and further research programs for stroke treatment.

**Methods:**

We searched the online database for paper published between January 2015 and April 2021. We calculated weighted mean difference (WMD) or odds risk (OR) and their corresponding 95% confidence interval (95% CI) of reported outcomes between HUK plus Eda and Eda groups for each study. The random‐effect models or fixed‐effect models were used to pool the analysis.

**Results:**

Thirteen studies with 1242 patients were included. In the pooled analysis, the scores of NIHSS in the HUK plus Eda group were significantly lower than that in patients receiving Eda (WMD = –3.92, 95% CI (–4.82, –3.02), *p *< .0001). The ADL scores in the HUK plus Eda group were significantly greater than that in patients receiving Eda (WMD = 14.13, 95% CI (10.67, 17.60), *p *< .0001). Furthermore, HUK plus Eda was associated with a higher rate of total efficacy (OR = 3.97, 95% CI (2.81, 5.59), *p *< .0001).

**Conclusions:**

HUK combined with Eda provides potential clinical benefits as a treatment for AIS. Further high‐quality, large‐scale randomized trials are needed to confirm these results.

## Graphical abstract

Our results provided further evidence that HUK combined with Eda provides potential clinical benefits as a treatment for AIS.

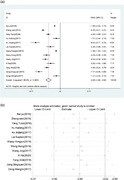

## 1 INTRODUCTION

Stroke is the second leading cause of death and a major cause of disability across the world (Katan & Luft, 2018). It is the leading cause of death and disability in China, which accounts for a fifth of the world's population (Wu et al., 2019). To meet this challenge, the China Stroke Prevention Project Committee (CSPPC) of the Ministry of Health was established in April 2011. This committee actively promotes stroke prevention and control in China (Chao et al., 2021). Acute ischemic stroke (AIS) is the most common type of stroke, which is caused by the blockage of a blood vessels supplying to the brain, accounting for about 80% of all varieties of stroke (Wang et al., 2015). Recanalization, especially thrombolysis, can significantly improve outcomes. However, hemorrhagic transformation, neurotoxicity, and a short treatment time window are major limitations of thrombolytic therapy (Bennink et al., 2015; Ishiguro et al., 2012).

Human urinary kallidinogenase (HUK) is a glycoprotein that is extracted from male urine. HUK belongs to the tissue kallikrein family, and tissue kallikreins exert their biological effects by activating kallikrein/kinin system (KKS). Activated KKS would induce therapeutic angiogenesis and neovascularization, which might provide a new way to restore blood supply in the ischemic area (Emanueli & Madeddu, 2004 , 2003; Han et al., 2015; Pérez et al., 2006). HUK treatment has been taken into consideration following the results of several randomized controlled trials (level IIB) (Neurology & Society, 2018). In addition, HUK has been approved by the State Food and Drug Administration of China and has been clinically used in the treatment of stroke patients in China for more than 10 years. HUK has been reported to promote angiogenesis, enhance cerebral perfusion, and suppress the inflammatory response in animal trials (Chen et al., 2010; Han et al., 2015). A study with animal models demonstrated that HUK can significantly improve neurological function with few adverse effects (Chen et al., 2010).

Meanwhile, edaravone (Eda) was first approved by the Japanese Ministry of Health for the treatment of ischemic stroke in 2001. It inhibits lipid peroxidation (Higashi et al., 2006), scavenges free radicals and oxidative damage to nerve cells, endothelial cells, and brain cells (Yoshida et al., 2006), and reduces the effects of cerebral ischemia and edema (Nakamura et al., 2008), thus reducing the tissue damage caused by acute cerebral infarction.

HUK and Eda are a new selective cerebral vasodilator and oxygen‐free radical scavenger, which were widely used in the treatment of cerebral infarction in the past few years. In recent years, a series of studies have compared the efficacy of HUK combined with Eda in the treatment of acute stroke (Aojinjiang, 2017; Haibing, 2017; Hong, 2016; Hongjun, 2019; Menglin, 2017). In the present study, we performed a meta‐analysis assessing the efficacy of HUK combined with Eda in treating AIS. Our aim was to provide the best available evidence for clinical practice and further research programs for stroke treatment.

## 2 METHODS

This meta‐analysis was reported according to the Preferred Reporting Items for Systematic Reviews and Meta‐Analysis (PRISMA) guidelines (Shamseer et al., 2015). All analyses were based on previously published studies; thus, ethical approval or patient consent was not suitable for this meta‐analysis.

### 2.1 Data sources and searches

Systematic literature searches using specific keywords were performed on Chinese databases, including Wanfang and China National Knowledge Infrastructure (CNKI), and English literature database (PubMed, Web of science, and Embase) from January 2015 to April 2021. The combinations of search keywords used to conduct our search were “Human Urinary Kallidinogenase,” “Urinary Kallikrein,” “HUK,” “Edaravone,” “Eda,” “acute ischemic stroke,” “stroke,” “Acute stroke,” “Acute cerebral infarction,” and “cerebral infarction.” All records were searched by two researchers separately, and all articles that could possibly satisfy the inclusion criteria according to one of the researchers were retrieved as full text. The decision to include or exclude a study was also made by two independent researchers. Disagreements were solved through discussion.

### 2.2 Study selection and data search

We identified studies that reported clinical outcomes in patients treated with HUK combined with Eda and control group with Eda. The inclusion criteria of studies were as following: (1) randomized controlled design; (2) studies comparing efficacy of HUK plus Eda versus Eda in patients with AIS; (3) reporting of clinical outcome data including neurophysiological outcomes, functional outcomes, and total efficacy rate. Neurophysiological outcomes were assessed by the National Institutes of Health Stroke Scale (NIHSS) and the functional outcomes were assessed by the activities of daily living (ADL). The exclusion criteria of studies were: (1) studies were excluded in which the means and SDs (or number (%)) were not reported; (2) duplicated studies, case report, review, abstracts, conference and systematic reviews, and meta‐analysis; (3) intervention of control group was not Eda.

We extracted the following information: participant characteristics (i.e., age, clinical outcomes, and target group), first author and year of publication, sample size, interventions, and follow‐up duration. The outcomes included an assessment of neurological improvement in the NIHSS and an assessment of ADL improvement and total efficacy rate.

### 2.3 Risk of bias assessment

We assessed the validity of included studies according to the Cochrane Collaboration's tool, which contains seven domains: random sequence generation, allocation concealment, blinding of participants and personnel, blinding of outcome assessment, incomplete outcome data, selective reporting, and other sources of bias. Disagreements were solved through discussion.

### 2.4 Statistical analysis

We calculated weighted mean difference (WMD) or odds risk (OR) and their corresponding 95% confidence interval (95% CI) of reported outcomes between HUK plus Eda and Eda groups for each study. As an estimate of the heterogeneity of effect sizes, we calculated the *I* ^2^ statistic and Q test. According to the result of heterogeneity, random‐effect models or fixed‐effect models were used to pool the analysis. We used Egger test to examine publication bias and to examine whether it was statistically significant. Sensitivity analyses were used to test the robustness of the pooled effect. All statistical analyses were developed with Stata15.0 software.

## 3 RESULTS

### 3.1 Characteristics of studies

**FIGURE 1:**
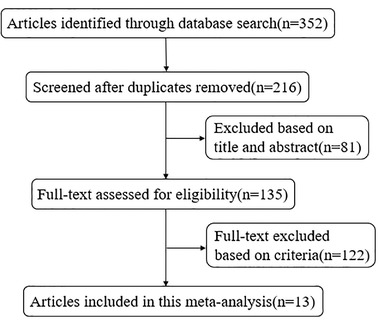
Flowchart of search strategy

After screening a total of 216 titles and abstracts (136 after removal of duplicates), we screened 135 full‐text studies for further consideration and excluded 122 studies. The flowchart describing the inclusion process is showed in Figure 1. Finally, 13 studies with 1242 patients (treatment group with 632, control group with 610) satisfied the inclusion criteria. Baseline characteristics of the included studies are presented in the Table 1. The average age of patients in the included studies was over 50 years old. The doses of HUK and Eda were 0.15 PNA/day, 30 mg/day, respectively. The mean values for clinical outcomes were assessed at 14 days.

**TABLE 1:** Baseline characteristic of the included studies

Author	Sampling, T/C	Age, T/C	Intervention, T/C	Duration, outcomes
Bai et al. (Yu., 2016)	58/52	58.55.±.7.02 61.78 ± 9.01	UK.+.E,.0.15 PNA, 1/day E, 30 mg, 2/day	14 days, NIHSS, ADL, Total effective rate
Zhang et al. (Wen, 2019)	54/54	62.34 ± 4.98 62.28 ± 4.76	UK + E,0.15 PNA, 1/day E,30 mg, 1/day	14 days, NIHSS, ADL, Total effective rate
Yang et al. (Yubo, 2016)	47/47	— —	UK + E, 0.15 PNA, 1/day E, 30 mg, 1/day	14 days, NIHSS, ADL, Total effective rate
Hu et al. (Haibing, 2017)	45/45	61.73 ± 6.59 61.25 ± 6.40	UK + E, 0.15U, 1/day E, 30 mg, 1/12h	14 days, NIHSS, ADL, Total effective rate
Ao et al. (Aojinjiang, 2017)	45/45	62.30 ± 7.20 65.30 ± 7.80	UK + E,0.15 PNA,1/day E, 30 mg, 2/day	14 days, NIHSS, ADL, Total effective rate
Lai et al. (Suiping, 2015)	34/33	65.01 ± 10.72 59.65 ± 8.91	UK + E, 0.15 PNA, 1/day E, 30 mg, 2/day	14 days, NIHSS, ADL, Total effective rate
Wang et al. (Hongjun, 2019)	58/58	63.47 ± 4.15 62.42 ± 4.17	UK + E, 0.15 mg, 1/day E, 30 mg, 2/day	14 days, NIHSS, ADL, Total effective rate
Wang et al. (Hong, 2016)	48/48	60.39 ± 8.23 61.05 ± 8.29	UK + E, 0.15 PNA, 1/day E, 30 mg, 1/day	14 days, NIHSS, ADL, Total effective rate
Wang et al. (Wang Jing, 2017)	50/50	— —	UK + E, 0.15 mg, 1/day E, 30 mg, 2/day	14 days, NIHSS, ADL, Total effective rate
Xi et al. (Na, 2020)	56/41	63.80 ± 11.40 67.50 ± 10.20	UK + E, 0.15 mg, 1/day E, 30 mg, 2/day	14 days, NIHSS, ADL, Total effective rate
Yang et al. (Xinli, 2019)	57/57	68.20 ± 3.30 68.80 ± 3.10	UK + E,0.15 PNA, 1/day E,3 0 mg, 1/day	14 days, NIHSS, ADL, Total effective rate
Jiang et al. (Bingquan, 2016)	35/35	63.34 ± 2.56 63.74 ± 2.48	UK + E, 0.15 PNA, 2/day E,0.03 g, 2/day	14 days, NIHSS, ADL, Total effective rate
Deng et al. (Menglin, 2017)	45/45	61.80 ± 6.70 61.40 ± 6.41	UK + E, 0.15 PNA, 1/day E, 0.03 g, 2/day	14 d, NIHSS, ADL, Total effective rate

*Abbreviations*: ADL, activities of daily living; C, control; E, edaravone; NIHSS, National Institutes of Health Stroke Scale; T, treatment; UK + E, urinary kallidinogenase + edaravone.

**FIGURE 2:**
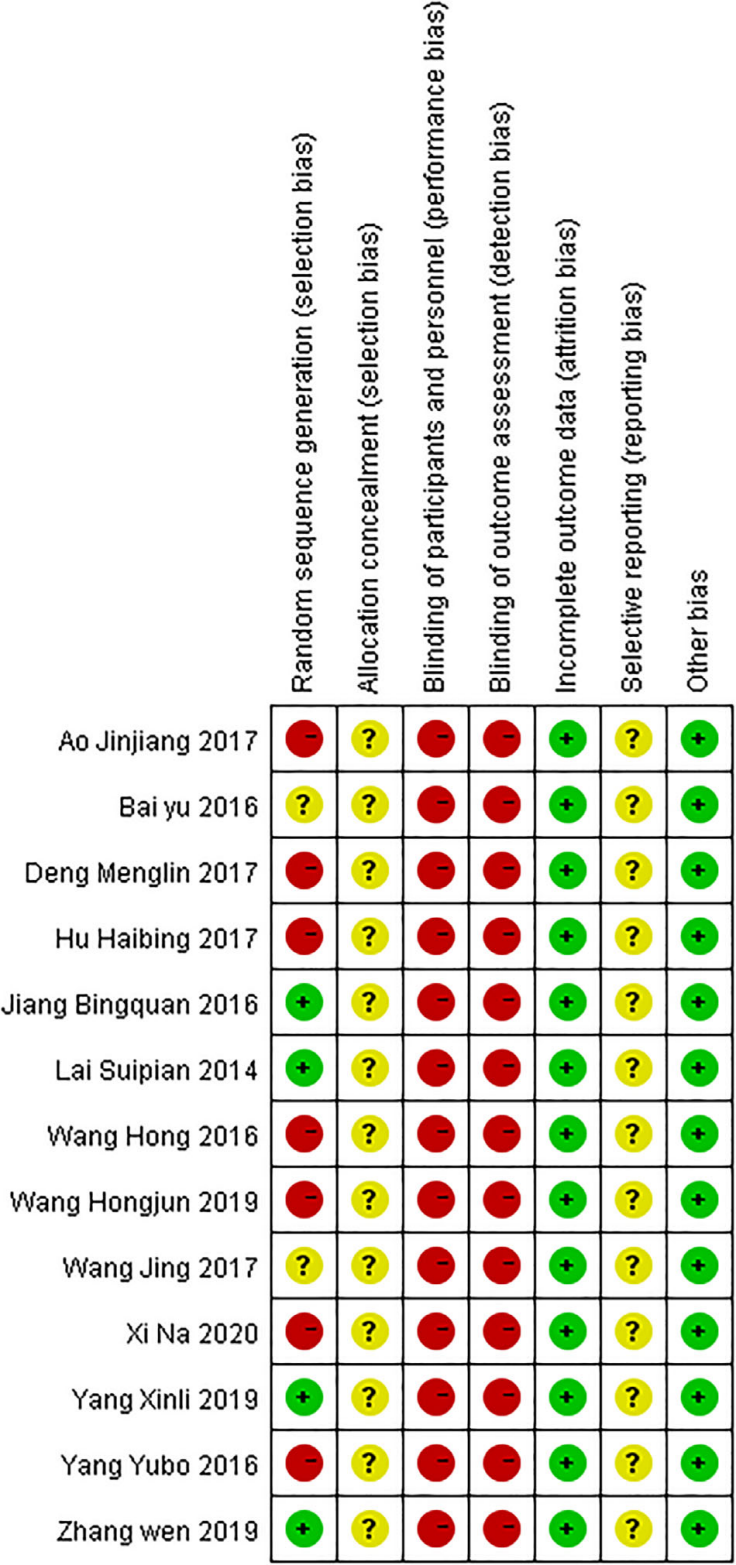
Risk of bias assessment

With a regard to risk of bias, four studies (30.76%) reported an adequate random sequence generation; allocation concealment were not described for all studies; all included studies did not report blinding of participants and outcome assessment. All the studies were considered to have a high risk of performance bias due to their deficiency of blinding design (shown in Figure 2).

### 3.2 NIHSS

**FIGURE 3:**
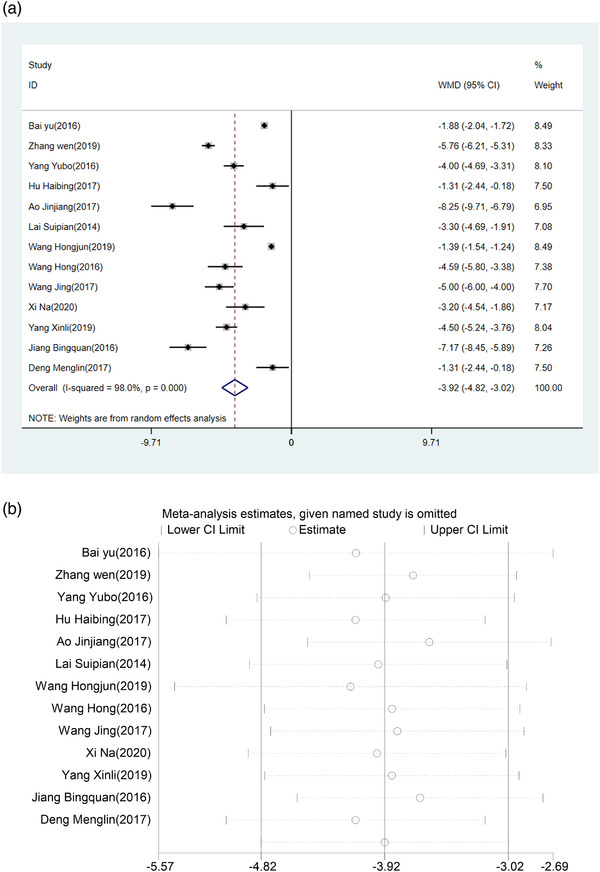
(a) Forest plot of NIHSS. (b) Sensitivity analysis of NIHSS

The random‐effect model revealed that the NIHSS score of patients treated with HUK combined with Eda was lower than that of patients treated with Eda alone, and the difference was statistically significant (WMD = −3.92, 95% CI (−4.82, −3.02), *p *< .0001), with high evidence of the heterogeneity(*I* ^2 ^= 98.0%, *p *< .001) (Figure 3a). The neurological function was better in the HUK combined with Eda group than in the Eda alone group. Sensitivity analysis showed that pooled effect changed slightly by removing each study one at a time (Figure 3b).

### 3.3 ADL

**FIGURE 4:**
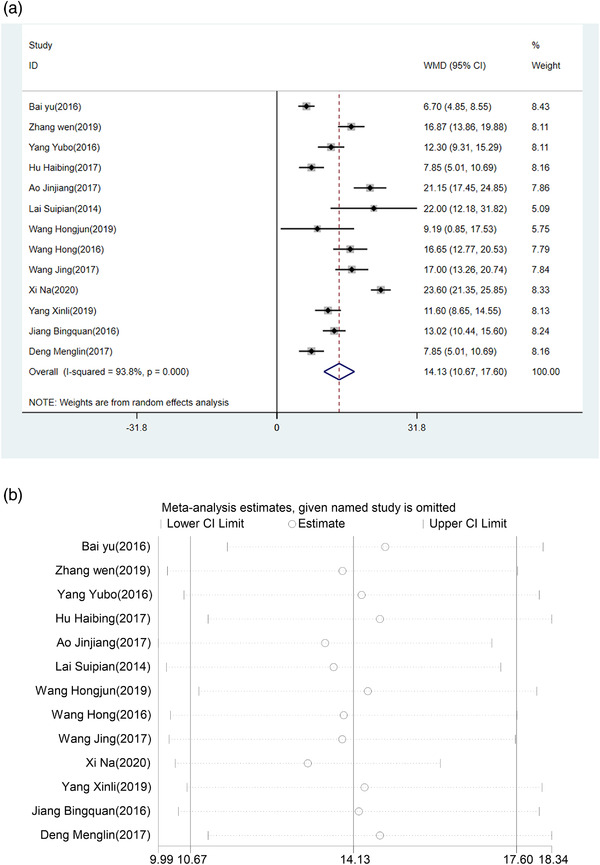
(a) Forest plot of ADL. (b) Sensitivity analysis of ADL

The result of pooled effect by fixed‐effect model showed that compared with Eda alone group, the ADL score of the HUK combined with Eda group was significantly higher (WMD = 14.13, 95% CI (10.67, 17.60), *p *< .0001), with considerable heterogeneity among studies (*I* ^2 ^= 93.8%, *p *< .0001) (Figure 4a). The ADL was an improvement in the HUK plus Eda group than in the Eda alone group. According to the sensitivity analysis, the result was robust as shown in Figure 4b.

### 3.4 Total efficacy rate

**FIGURE 5:**
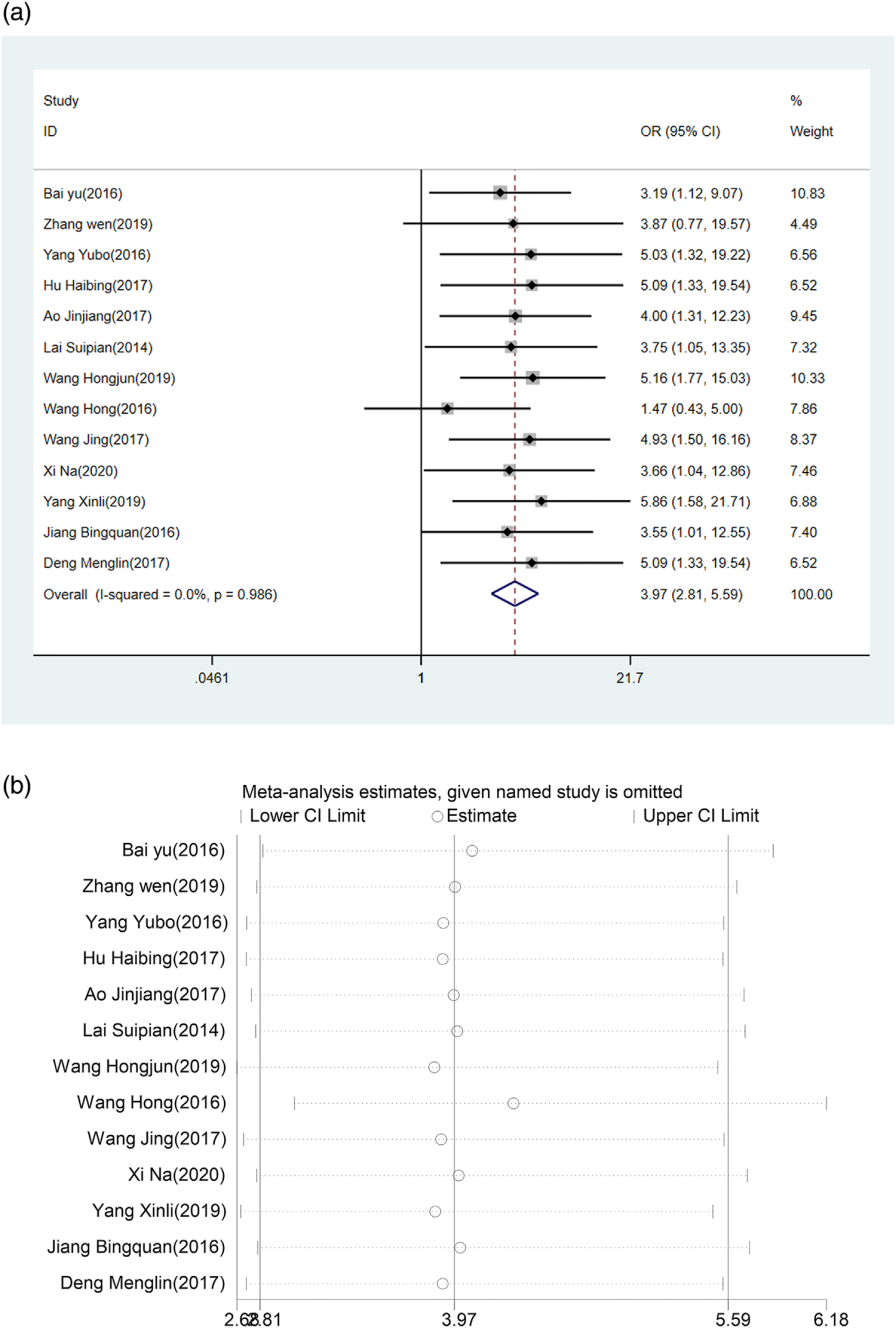
(a) Forest plot of total efficacy rate. (b) Sensitivity analysis of total efficacy rate

Pooled effects by a fixed‐effects model demonstrated that HUK combined with Eda treatment was associated with a high total efficacy rate (OR = 3.97, 95% CI (2.81, 5.59), *p *< .0001), with no heterogeneity across studies (*I* ^2 ^= 0.0%, *p *= .986) (Figure 5a). According to the sensitivity analysis, the result was robust as shown in Figure 5b. According to the Egger’ test (*p *= .665), little publication bias was discovered among the studies.

## 4 DISCUSSION

The purpose of this meta‐analysis was to assess the efficacy of HUK combined with Eda in the treatment of AIS. Our results indicated that patients treated with HUK combined with Eda significantly decreased the NIHSS score compared with baseline NIHSS score, improved the ADL score compared with baseline ADL score, and had a better total efficacy rate than that of patients treated with Eda alone. A meta‐analysis by Huang et al. (2020) that included 16 trials compared the efficacy and safety between the HUK treatment group and basic treatment. The results revealed that HUK had more neurological improvement than the basic treatment groups in NIHSS scores (mean difference: −1.65, 95% CI (−2.12, −1.71)) and clinical efficacy. The study also found that adverse effects were no different between the HUK group and basic treatment group(Huang et al., 2020). A systematic review by Zhang et al. (2012) assessed the efficacy and safety of HUK for AIS. Trails were included for patients with HUK versus placebo or no intervention. The results suggested that HUK appears to reduce neurological impairment for patients with AIS and to improve long‐term outcomes (Zhang et al., 2012). Compared with these studies, our study assessed the efficacy of HUK combined with Eda in the treatment of AIS, and the control group was Eda alone. Our study not only evaluated the neurological function and effectiveness, but also evaluated the impact on the ADL function. And our results were consistent with previous studies. HUK‐combined Eda may be regarded as a potential choice for the treatment of AIS patients.

In the studies of combined medicine, HUK combined with butylphthalide can improve the long‐term independency rate of AIS patients, and the efficacy of HUK‐combined therapy is better than that of Eda (Qian et al., 2019). Patients of massive cerebral infarction treated with urinary kallidinogenase‐combined Eda has a certain curative effect (Ke & Jing, 2016). A prospective study with 58 stroke patients confirmed that HUK promoted stroke recovery, enhanced cerebral reperfusion through up‐regulating vascular endothelial growth factor, apelin/APJ pathway, and the average perfusion time, was significantly shortened (Li et al., 2015). According to the trail of Org 10172 in Acute Stroke Treatment classification, HUK can significantly improve the NIHSS score of cerebral ischemia caused by large‐artery atherosclerosis and small‐artery atherosclerosis and it is helpful in improving the clinical efficacy (Li et al., 2015). A study using magnetic resonance perfusion weighted imaging (MRP) methods evaluated the microcirculation and concluded that HUK could enhance cerebral blood flow in the lesioned hemisphere, but not decrease the cerebral blood flow of the contralateral hemisphere (Miao et al., 2016). The benefit of HUK promotes brain remodeling, which might be an important mechanism in the treatment of acute cerebral infarction (Miao et al., 2016). According to a study Song et al. (2012), HUK induced over activation of ipsilateral primary motor sensory cortex compared with the control group, suggesting that HUK improved more nerve fibers and/or restored more innervation at the injured site after ischemia. Meanwhile, HUK treatment can enhance the activation of ipsilateral auxiliary motor area, premotor cortex, and contralateral posterior parietal cortex, indicating that HUK can regulate motor function reconstruction in patients with cerebral infarction. Kallikrein kinin system can be activated by HUK (Sahan et al., 2013), hydrolyze kininogen into kinin, and release nitric oxide (NO) to relax vascular smooth muscle (Perilli et al., 2012). In addition, kinin as another component of kallikrein kinin system can reduce the expression of vascular endothelial growth factor and its receptor, thus promoting angiogenesis (Ke & Jing, 2016). These findings may explain the mechanism of HUK in preventing stroke recurrence.

Some limitations of this meta‐analysis should be noted. First, all included studies were published in Chinese, this may cause a limitation of general applicability. Second, the overall methodological quality of the included studies was not ideal, especially the short of double‐blind design. There were some heterogeneities among the included studies, although the random‐effect model was used to solve this problem. Further high‐quality randomized controlled trials of the clinical efficacy of HUK combined with Eda for AIS are needed.

## 5 CONCLUSIONS

Our results indicated that the scores of NIHSS in the HUK plus Eda group were significantly lower than that in patients receiving Eda, and ADL scores were significantly greater than that in patients receiving Eda. Our findings suggested that HUK‐combined Eda can improve the nerve function and ADL function. The effectiveness of HUK‐combined Eda therapy is better than Eda. It may be regarded as a potential choice for the treatment of AIS patients. Further high‐quality, large‐scale randomized trials are needed to confirm these results.

## CONFLICT OF INTEREST

The authors declare that they have no conflict of interest.

## AUTHOR CONTRIBUTIONS

Dan Yu designed the literature search and analysis. Yao Li, Bi Guan, and Qian Ming searched the studies. Yan Li and Li‐Qing Chen performed the quality assessment of the results. Di‐Xiao Yang and Yao Li analyzed the data and interpreted the result. Di‐Xiao Yang wrote the manuscript. Dan Yu and Di‐Xiao Yang finalized the manuscript. All authors discussed, reviewed, and approved the final manuscript.

## FUNDING INFORMATION

The authors received no funding for this work.

### PEER REVIEW

The peer review history for this article is available at https://publons.com/publon/10.1002/brb3.2431

## Data Availability

Data availability statement is not applicable to meta‐analysis.

## References

[brb32431-bib-0001] Aojinjiang, H. J. , & Song, L. (2017). Eureklin and edaravone on the improvement of cerebral vascular reserve and the protection of nerve function in patients with acute cerebral infarction. Journal of Hunan Normal University, 5, 144–147.

[brb32431-bib-0002] Bennink, E. , Horsch, A. D. , Dankbaar, J. W. , Velthuis, B. K. , Viergever, M. A. , & de Jong, H. W. (2015). CT perfusion analysis by nonlinear regression for predicting hemorrhagic transformation in ischemic stroke. Medical Physics, 42(8), 4610–4618. 10.1118/1.4923751 26233188

[brb32431-bib-0003] Bingquan, J. (2016). Clinical effect observation of urinary kallidinogenase combined with edaravone in the treatment of acute cerebral infarction. Clinical Medicine, 56–58.

[brb32431-bib-0004] Chao, B. H. , Yan, F. , Hua, Y. , Liu, J. M. , Yang, Y. , Ji, X. M. , Peng, B. , Zhao, G. G. , Wang, Y. J. , Kang, D. Z. , Wang, Y. L. , Zeng, J. S. , Chu, L. , Li, T. X. , Xu, Y. M. , Liu, M. , He, L.i , Xu, Y. , Wu, J. , … Wang, L. D. (2021). Stroke prevention and control system in China: CSPPC‐Stroke Program. International Journal of Stroke, 16(3), 265–272. 10.1177/1747493020913557 32223541

[brb32431-bib-0005] Chen, Z. B. , Huang, D. Q. , Niu, F. N. , Zhang, X. , Li, E. G. , & Xu, Y. (2010). Human urinary kallidinogenase suppresses cerebral inflammation in experimental stroke and downregulates nuclear factor‐kappaB. Journal of Cerebral Blood Flow and Metabolism, 30(7), 1356–1365. 10.1038/jcbfm.2010.19 20179726 PMC2949229

[brb32431-bib-0006] Emanueli, C. , & Madeddu, P. (2003). Human tissue kallikrein: A new bullet for the treatment of ischemia. Current Pharmaceutical Design, 9(7), 589–597. 10.2174/1381612033391315 12570806

[brb32431-bib-0007] Emanueli, C. , & Madeddu, P. (2004). Angiogenesis therapy with human tissue kallikrein for the treatment of ischemic diseases. Archives Des Maladies Du Coeur Et Des Vaisseaux, 97(6), 679–687. 15283043

[brb32431-bib-0008] Haibing, Z. L. H. , & Lihaipeng, F. G. (2017). Clinical observation of the combination of yuruiklin and edaravone in the treatment of acute ischemic stroke. Contemporary Medicine Forum, 023(021), 96–98.

[brb32431-bib-0009] Han, L. , Li, J. , Chen, Y. , Zhang, M. , Qian, L. , Chen, Y. , Wu, Z. , Xu, Y. , & Li, J. (2015). Human urinary kallidinogenase promotes angiogenesis and cerebral perfusion in experimental stroke. PLOS One, 10(7), e0134543. 10.1371/journal.pone.0134543 26222055 PMC4519127

[brb32431-bib-0010] Higashi, Y. , Jitsuiki, D. , Chayama, K. , & Yoshizumi, M. (2006). Edaravone (3‐methyl‐1‐phenyl‐2‐pyrazolin‐5‐one), a novel free radical scavenger, for treatment of cardiovascular diseases. Recent Patents on Cardiovascular Drug Discovery, 1(1), 85–93. 10.2174/157489006775244191 18221078

[brb32431-bib-0011] Hong, L. H. W. (2016). Efficacy of urinary kallidinogenase combined with edaravone in the treatment of acute cerebral infarction and its dynamic effect on CRP. China Practical Medicine, 11(3), 121–122.

[brb32431-bib-0012] Hongjun, L. C. W , & Gong, Z. (2019). Analysis of clinical value of urinary kallidinogenase combined with edaravone in the treatment of acute progressive cerebral infarction. Practical Journal of Clinical Medicine, 16(6), 154–157.

[brb32431-bib-0013] Huang, Y. , Wang, B. , Zhang, Y. , Wang, P. , & Zhang, X. (2020). Efficacy and safety of human urinary kallidinogenase for acute ischemic stroke: A meta‐analysis. Journal of International Medical Research, 48(9), 300060520943452. 10.1177/0300060520943452 PMC7780570 32954870

[brb32431-bib-0014] Ishiguro, M. , Kawasaki, K. , Suzuki, Y. , Ishizuka, F. , Mishiro, K. , Egashira, Y. , Ikegaki, I. , Tsuruma, K. , Shimazawa, M. , Yoshimura, S. , Iwama, T. , & Hara, H. (2012). A Rho kinase (ROCK) inhibitor, fasudil, prevents matrix metalloproteinase‐9‐related hemorrhagic transformation in mice treated with tissue plasminogen activator. Neuroscience, 220, 302–312. 10.1016/j.neuroscience.2012.06.015 22710066

[brb32431-bib-0015] Jing, J. Z. W (2017). Efficacy of urinary kallidinogenase combined with edaravone in the treatment of acute cerebral infarction and its dynamic effect on CRP. Modern Diagnosis and Treatment, 17, 3179–3180.

[brb32431-bib-0016] Katan, M. , & Luft, A. (2018). Global burden of stroke. Seminars in Neurology, 38(2), 208–211. 10.1055/s-0038-1649503 29791947

[brb32431-bib-0017] Ke, J. , & Jing, M. (2016). Analysis of treatment effect of urinary kallidinogenase combined with edaravone on massive cerebral infarction. Biomedical Reports, 5(2), 155–158. 10.3892/br.2016.692 27446533 PMC4950744

[brb32431-bib-0018] Li, J. , Chen, Y. , Zhang, X. , Zhang, B. , Zhang, M. , & Xu, Y. (2015). Human urinary kallidinogenase improves outcome of stroke patients by shortening mean transit time of perfusion magnetic resonance imaging. Journal of Stroke & Cerebrovascular Diseases, 24(8), 1730–1737. 10.1016/j.jstrokecerebrovasdis.2015.03.032 26139453

[brb32431-bib-0019] Li, C. , Zha, O. G. , He, Q. Y. , Wu, Y. Z. , Wang, T. S. , & Teng, J. F. (2015). Study on the clinical efficacy of Human Urinary Kalllikrein in the treatment of acute cerebral infarction according to TOAST classification. Pakistan Journal of Pharmaceutical Sciences, 28(4), 1505–1510. 26431650

[brb32431-bib-0020] Menglin, D. (2017). Clinical observation of urinary kallidinogenase combined with edaravone in the treatment of acute ischemic stroke. Chinese And Foreign Medical Research, 15(20), 133–134.

[brb32431-bib-0021] Miao, J. , Deng, F. , Zhang, Y. , Xie, H. Y. , & Feng, J. C. (2016). Exogenous human urinary kallidinogenase increases cerebral blood flow in patients with acute ischemic stroke. Neurosciences, 21(2), 126–130. 10.17712/nsj.2016.2.20150581 27094522 PMC5107266

[brb32431-bib-0022] Na, X. (2020). Urinary kallidinogenase combined with edaravone in the treatment of acute cerebral infarction. Chinese Medical Journal of Metallurgical Industry, 37(06).

[brb32431-bib-0023] Nakamura, T. , Kuroda, Y. , Yamashita, S. , Zhang, X. , Miyamoto, O. , Tamiya, T. , Nagao, S. , Xi, G. , Keep, R. F. , & Itano, T. (2008). Edaravone attenuates brain edema and neurologic deficits in a rat model of acute intracerebral hemorrhage. Stroke, 39(2), 463–469. 10.1161/strokeaha.107.486654 18096835

[brb32431-bib-0024] Neurology branch of Chinese Medical Association, cerebrovascular disease group of Neurology branch of Chinese Medical Association . (2018). Chinese guidelines for the diagnosis and treatment of acute ischemic stroke 2018. Chinese Journal of Neurology, 51(9), 666–682.

[brb32431-bib-0025] Pérez, V. , Leiva‐Salcedo, E. , Acuña‐Castillo, C. , Aravena, M. , Gómez, C. , Sabaj, V. , Colombo, A. , Nishimura, S. , Pérez, C. , Walter, R. , & Sierra, F. (2006). T‐kininogen induces endothelial cell proliferation. Mechanisms of Ageing and Development, 127(3), 282–289. 10.1016/j.mad.2005.11.002 16378635

[brb32431-bib-0026] Perilli, V. , Aceto, P. , Modesti, C. , Ciocchetti, P. , Sacco, T. , Vitale, F. , Lai, C. , Magalini, S. C. , Avolio, A. W. , & Sollazzi, L. (2012). Low values of left ventricular ejection time in the post‐anhepatic phase may be associated with occurrence of primary graft dysfunction after orthotopic liver transplantation: Results of a single‐centre case‐control study. European Review for Medical and Pharmacological Sciences, 16(10), 1433–1440. 23104662

[brb32431-bib-0027] Qian, Y. , Lyu, Y. , Jiang, M. , Tang, B. , Nie, T. , & Lu, S. (2019). Human urinary kallidinogenase or edaravone combined with butylphthalide in the treatment of acute ischemic stroke. Brain and Behavior, 9(12), e01438. 10.1002/brb3.1438 31638334 PMC6908872

[brb32431-bib-0028] Sahan, M. , Sebe, A. , Acikalin, A. , Akpinar, O. , Koc, F. , Ay, M. O. , Gulen, M. , Topal, M. , & Satar, S. (2013). Acute‐phase reactants and cytokines in ischemic stroke: Do they have any relationship with short‐term mortality? European Review for Medical and Pharmacological Sciences, 17(20), 2773–2777. 24174359

[brb32431-bib-0029] Shamseer, L. , Moher, D. , Clarke, M. , Ghersi, D. , Liberati, A. , Petticrew, M. , Shekelle, P. , & Stewart, L. A. (2015). Preferred reporting items for systematic review and meta‐analysis protocols (PRISMA‐P) 2015: Elaboration and explanation. BMJ, 350, g7647. 10.1136/bmj.g7647 25555855

[brb32431-bib-0030] Song, X. , Han, L. , & Liu, Y. (2012). Remodeling of motor cortex function in acute cerebral infarction patients following human urinary kallidinogenase: A functional magnetic resonance imaging evaluation after 6 months. Neural Regeneration Research, 7(11), 867–873. 10.3969/j.issn.1673-5374.2012.11.012 25737716 PMC4342716

[brb32431-bib-0031] Suiping, L. (2015). Effect analysis of urinary kallidinogenase combined with edaravone in the treatment of massive cerebral infarction. Hebei Medical Journal, 7, 994–996.

[brb32431-bib-0032] Wang, H. R. , Chen, M. , Wang, F. L. , Dai, L. H. , Fei, A. H. , Liu, J. F. , Li, H. J. , Shen, S. , Liu, M. , & Pan, S. M. (2015). Comparison of therapeutic effect of recombinant tissue plasminogen activator by treatment time after onset of acute ischemic stroke. Scientific Reports, 5, 11743. 10.1038/srep11743 26206308 PMC4513278

[brb32431-bib-0033] Wen, Z. (2019). Clinical efficacy and safety of edaravone combined with urinary kallidinogenase in the treatment of patients with acute cerebral infarction. The Journal of Medical Theory and Practice, 32(17), 53–55.

[brb32431-bib-0034] Wu, S. , Wu, B. , Liu, M. , Chen, Z. , Wang, W. , Anderson, C. S. , Sandercock, P. , Wang, Y. , Huang, Y. , Cui, L. , Pu, C. , Jia, J. , Zhang, T. , Liu, X. , Zhang, S. , Xie, P. , Fan, D. , Ji, X. , Wong, K. S. L. , … Wang, L. (2019). Stroke in China: Advances and challenges in epidemiology, prevention, and management. Lancet Neurology, 18(4), 394–405. 10.1016/s1474-4422(18)30500-3 30878104

[brb32431-bib-0035] Xinli, Y. (2019). Clinical efficacy of urinary kallidinogenase combined with edaravone in the treatment of acute cerebral infarction. Modern Medical Imageology, 28(02), 438–439.

[brb32431-bib-0036] Yoshida, H. , Yanai, H. , Namiki, Y. , Fukatsu‐Sasaki, K. , Furutani, N. , & Tada, N. (2006). Neuroprotective effects of edaravone: A novel free radical scavenger in cerebrovascular injury. CNS Drug Reviews, 12(1), 9–20. 10.1111/j.1527-3458.2006.00009.x 16834755 PMC6741743

[brb32431-bib-0037] Yu, B. (2016). Clinical observation of edaravone combined with urinary kallidinogenase in the treatment of acute progressive cerebral infarction. World Latest Medicine Information, 65, 107–108.

[brb32431-bib-0038] Yubo, Y. (2016). Study on the effect of Edaravone and urinary kallidinogenase in the treatment of acute cerebral infarction. Contemporary Medicine Forum, 14(17), 112–113.

[brb32431-bib-0039] Zhang, C. , Tao, W. , Liu, M. , & Wang, D. (2012). Efficacy and safety of human urinary kallidinogenase injection for acute ischemic stroke: A systematic review. Journal of Evidence‐Based Medicine, 5(1), 31–39. 10.1111/j.1756-5391.2012.01167.x 23528118

